# Oral health aspects in sporadic and familial primary hyperparathyroidism

**DOI:** 10.4317/jced.59527

**Published:** 2022-05-01

**Authors:** Fabiana-Brandão-Pain Cardoso, Fábio-Wildson-Gurgel Costa, Carlos-Eduardo-Lopes Soares, Maria-Elisabete-Amaral de Moraes, Catarina-Brasil D’alva, Davi-de Sá Cavalcante, Adília-Mirela-Pereira Cid, Thyciana-Rodrigues Ribeiro, Ana-Rosa-Pinto Quidute

**Affiliations:** 1 Postgraduate Program in Pharmacology, Department of Physiology and Pharmacology, Federal University of Ceará, Fortaleza, Brazil; 2 Postgraduate Program in Dentistry, Federal University of Ceará, Fortaleza, Brazil; 3 Faculty of Medicine, Drug Research and Development Center (NPDM), Federal University of Ceará, Fortaleza, Brazil; 4 Department of Physiology and Pharmacology, Drug Research and Development Center (NPDM), Faculty of Medicine, Federal University of Ceará, Fortaleza, Brazil; 5 Faculty of Medicine, Department of Clinical Medicine, Federal University of Ceará, Fortaleza, Brazil

## Abstract

**Background:**

Primary hyperparathyroidism (pHPT) is the third most common endocrinopathy, affecting 1-3% of postmenopausal women, with a total incidence of 21.6 cases per 100,000 people in the adult population. This study aimed to analyze the oral health and related aspects of individuals with pHPT.

**Material and Methods:**

A cross-sectional observational study was carried out on 51 patients diagnosed with pHPT associated with multiple endocrine neoplasia type 1 (MEN-1) (G1) or sporadic pHPT (G2). The oral aspects investigated were periodontal parameters, salivary flow, presence of dental caries, number of restored or missing teeth, and presence of tori. The biochemical parameters were collected in periods close to the dental evaluation.

**Results:**

In G1, 29 individuals (19 females) aged 40.24±13.06 years were included; in G2, 22 individuals (21 females) aged 64.09±10.01 years were included. Grade 2 mobility (*p*=0.031), mean probing depth (*p*<0.001), loss of clinical insertion level (*p*<0.001), gingival bleeding (*p*=0.009), and presence of palatine tori (*p*=0.007) were higher in G1. A higher mean of tooth loss (17.90±13.42; *p*=0.031), teeth with active and/or inactive caries (*p*<0.001), and visual change in enamel/enamel breakdown (*p*<0.001) were also observed in G1. Most patients were 50 years old or younger, with a higher prevalence of older individuals in G2 (*p*<0.001). G1 showed low socioeconomic status and G2 medium-high status (*p*<0.001).

**Conclusions:**

Despite the greater number of younger individuals, higher tooth loss and periodontal changes were observed in G1 patients. Differences in the degree of severity of pHPTor socioeconomic status alone could not explain these findings.

** Key words:**Oral health, Primary hyperparathyroidism, Multiple endocrine neoplasia type 1, osteopenia, osteoporosis.

## Introduction

The parathyroid glands are responsible for the secretion of the parathyroid hormone (PTH), which is involved in the regulation of calcium and phosphorus metabolism in the plasma and the bones. In addition, PTH plays an essential role in tooth development, bone mineralization and bone resorption (1). Primary hyperparathyroidism (pHPT) in its sporadic form affects in general 1-3% of women in the postmenopausal period, and it has an overall incidence of 21.6 cases per 100,000 individuals (2). pHPT may also have a genetic etiology in the pHPT associated with multiple endocrine neoplasia type 1 (MEN-1).

Intraoral disease has been described in patients with pHPT. The most common intraoral manifestations of pHPT have been described as loss of bone density in the maxillomandibular area, dental abnormalities, occlusion disorders, varying degrees of loss of tooth structure, tooth eruption disturbances, large pulp chamber, and brown tumor (3). In addition to these changes, the results of a study with pHPT patients suggested that high levels of PTH cause a reduction of the mandibular cortical bone, and its anabolic effect, combined with biomechanical forces of the oral cavity, allows the expansion of the trabecular bone. This trabecular expansion might explain the increased prevalence of maxillary tori observed in patients with this condition (4).

In this scenario, the study of systemic osteometabolic diseases can be considered a fertile field of research, with a strong correlation between medicine and dentistry, since several endocrine disorders exhibit manifestations in the maxillomandibular complex. This approach could help dentists identify these signs and help establish an early diagnosis of these disorders (5). Thus, this study can be justified on the fact that pHPT is one of the most frequent endocrine disorders around the world, with scarce data on oral manifestations in pHPT patients as well as limited research correlating these oral findings with metabolic and sociodemographic data.

## Material and Methods

-Ethical aspects, type of study, sample, and eligibility criteria

This study was approved by the Research Ethics Committee of the Federal University of Ceará (UFC) under protocol #2,206,456, following the Declaration of Helsinki. A cross-sectional observational study was carried out on patients diagnosed with pHPT/MEN-1 or sporadic pHTP from the sector of Clinical Endocrinology and Diabetes of the Walter Cantídio University Hospital (HUWC) (Fortaleza-CE), between August and November 2017. pHPT was defined as hypercalcemia with inappropriately high PTH level. Two study groups were defined: group 1 (G1) included volunteers with pHPT/MEN-1 (n=30); group 2 (G2) included individuals with sporadic pHPT (n=18). The included patients were clinically evaluated at the dental office of the Center for Drug Research and Development of the UFC.

This research included patients with pHPT/MEn-1 or sporadic pHTP of both sexes, aged over 18 years in periodic monitoring at the clinic of Endocrinology and Diabetes of the HUWC and who agreed to participate in the study after reading and signing a consent form. The diagnostic criterion for MEN-1 was clinical, characterized by the occurrence of tumors involving two or more endocrine glands. Voluntary participants who met at least one of the following criteria were excluded: hypercalcemia and/or elevated PTH levels unrelated to pHPT; periodontal treatment in the past six months; pregnancy, lactating, or use of oral contraceptives; smokers; alcohol and/or illicit drug users; use of medications capable of increasing plasma calcium concentration such as lithium, vitamin D, calcium supplementation or diuretic drugs in unusual dosage.

-Oral hygiene and salivary flow

Initially, all volunteers were submitted to anamnesis. Subsequently, a standardized saliva collection was performed between 8:00 and 11:00 am. The salivary flow of the unstimulated whole saliva was determined by the volume deposited in a 10 ml beaker for 5 minutes. The salivary flow was categorized as very low (<0.1 mL/min), low (>0.1 mL to 0.19 mL/min) and normal (≥2.0 mL/min).

-Examination of the oral cavity and evaluation of dental caries

The presence of soft and hard tissue changes was assessed by direct inspection of the oral cavity. The evaluation of dental caries followed the International Caries Detection and Assessment System (ICDAS), which was developed by an international team of caries researchers to integrate several new criteria systems in a standard system for the detection and evaluation of caries. The clinical examination was based on the codes for caries lesions defined by the ICDAS, investigating the lesion activity (active/inactive), affected tooth surface (mesial or distal), and classification scores for the identified lesions.

-Periodontal examination

The oral examination included the evaluation of the following periodontal parameters, according to the methodology used by Padbury *et al*. (2006) (4) and adapted to the present research: change in gingival color, change in gingival contour, change in gingival texture, tooth hypersensitivity, presence of gingival hyperplasia, presence of gingival recession, presence of mucosal injury, suppuration, presence and degree of tooth mobility, gingival bleeding index, plaque index, probing depth, clinical insertion level, and gingival margin level. The methodologies proposed by Silness, Löe (1964) (6) and Löe (1967) (7) were adopted to record dental plaque index and gingival bleeding index, respectively. Data on the clinical insertion level were collected according to the methodology described by Ainamo, Bay (1975) (8).

-Dental status

Panoramic radiographs of the patients were used to evaluate the dental status. The images were obtained using a Kodak 9000 3D CBCT scanner (kodak Dental Systems, Carestream Health, Toronto, Canada). The presence of endodontic treatment as well as the number of missing teeth in the maxilla and the mandible were analyzed. Dental functional status was classified based on the protocol proposed by Sato *et al*. (2016) (9) according to the number of natural teeth, with possible answers being ≥20, 10-19, 1-9, and no natural teeth (edentulous).

-Evaluation of biochemical, clinical, and sociodemographic aspects

Clinical and laboratory data used for diagnosis/monitoring of pHPT were collected from the patients’ medical records. Total calcium, ionized calcium, phosphorus, PTH, alkaline phosphatase, albumin and creatinine levels were analyzed from blood samples of the patients after overnight fasting under a free diet, and 24-h urinary calcium levels were measured in 24-h urine collections. These exams were performed in proximity with the dental appointment. In addition, a structured questionnaire designed for this research collected information reported in the hospital records on the following variables: age at diagnosis of pHPT, diagnosis of osteoporosis/osteopenia , age at diagnosis of osteoporosis/osteopenia, presence of bone fractures, age at bone fracture, presence of diabetes mellitus, and use of oral calcium or antiresorptive agents.

Sociodemographic data such as age, sex, place of birth, marital status, profession, socioeconomic status according to the Brazilian Criteria of Economic Classification (http://www.abep.org/criterio-brasil), family income (number of minimum wages), and years of education were also collected.

-Statistical analysis

The collected data were exported to the Statistical Package for the Social Sciences (SPSS) software (version 20.0) for Windows®, adopting a 95% confidence for the analyses. Quantitative data were expressed as means and standard deviation values and analyzed using the Student’s t-test, whereas the categorical data were expressed as absolute and percentage frequency values and analyzed using Fisher’s exact test and Pearson’s chi-square test. Dental variables were correlated with biochemical tests using Spearman’s correlation. Descriptive statistics (mean, median, and standard deviation) and data frequency were used. Statistical significance was considered when *p*<0.05.

## Results

-Participants

From an initial sample of 59 patients, 11 volunteers who met at least one of the following criteria were excluded: age under 18 years, smoker, or inconclusive diagnosis for pHPT. A total of 51 patients (40 women and 11 men) with pHPT (G1=29; G2=22) were included in this study. G1 had a mean age of 40.24±13.06 years, and the mean age in G2 was 64.09±10.01 years.

-Oral hygiene and salivary flow

The analysis of oral hygiene habits in both groups revealed that the most prevalent tooth brushing frequency was ≥3 times a day (56.3%). Most individuals mentioned the habit of night brushing (91.7%), but none of the volunteers mentioned flossing as a daily habit. The mean salivary flow was 0.21 ± 0.19 ml/min (ranging from 0.01 to 0.7 ml/min) in G1, and 0.16 ± 0.10 ml / min (ranging from 0.03 to 0.3 ml/min) in G2, with no statistically significant difference (*p*=0.898). None of the patients used hyposalivation-inducing drugs.

-Changes in the oral cavity, dental caries, and periodontal evaluation

The most significant finding in the examination of the soft tissues of the oral cavity was the occurrence of tori in 15.7% of all patients. In G1, this finding occurred in 27.6% of the cases, whereas, in G2, this occurrence was not observed (*p*=0.007). The maxilla was the most frequent location (75%) in comparison with the mandible (Table 1).

**Table 1:**
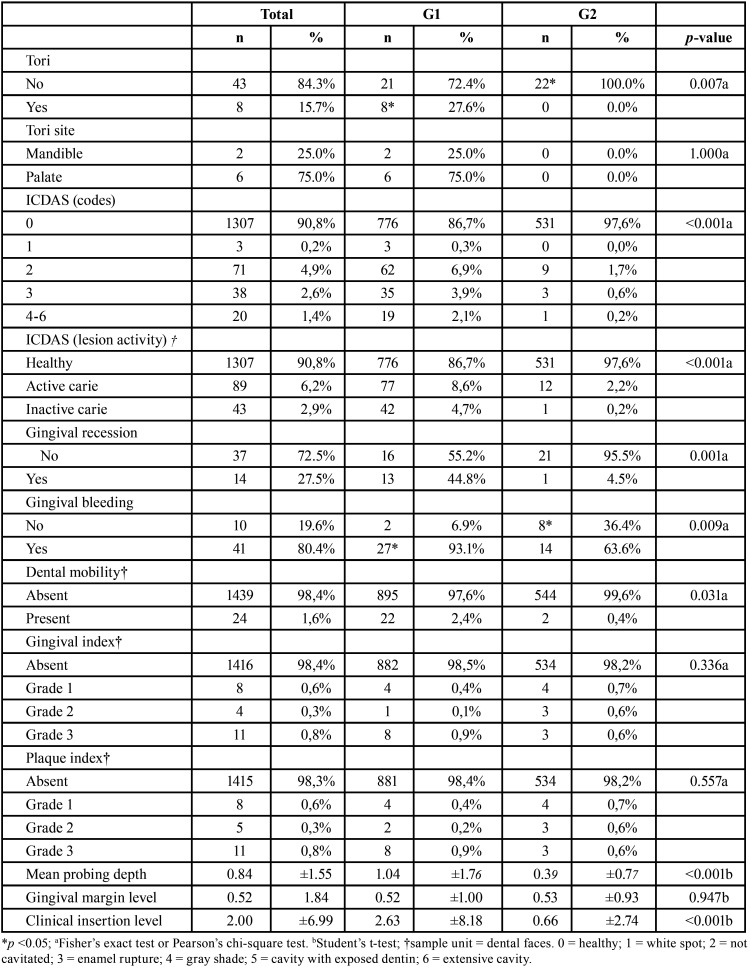
Comparison of oral health-related aspects between groups.

The evaluation of dental caries based on the ICDAS (Table 1) showed a statistically significant difference between the codes (*p*<0.001). G1 showed higher frequencies of codes 2 (distinct visual change in enamel), 3 (localized enamel breakdown), and 4-6 (codes denoting higher caries activity) compared to G2. Moreover, G1 had a higher frequency of teeth with caries regardless of the lesion activity (active or inactive) (*p*<0.001).

Regarding periodontal parameters (Table 1), the comparison between both groups revealed that G1 patients showed more gingival recession (*p*=0.001) and gingival bleeding (*p*=0.009) than in G2. Dental mobility was more frequently observed in G1 than in G2 (*p*=0.031). Moreover, mean probing depth (*p*<0.001) and clinical insertion level (*p*<0.001) were statistically higher in G1 than in G2.

-Tooth loss

In the analysis of dental radiographs, no suggestive signs of intraosseous osteolytic lesions were observed. Table 2 showed that most patients had ≤ 20 natural teeth; however, G1 patients showed a higher mean of tooth loss (17.90±13.42; *p*=0.031) and 55.2% individuals with >20 missing teeth (*p*=0.007). G2 demonstrated a higher frequency of upper teeth with signs of endodontic treatment (*p* <0.001).

**Table 2:**
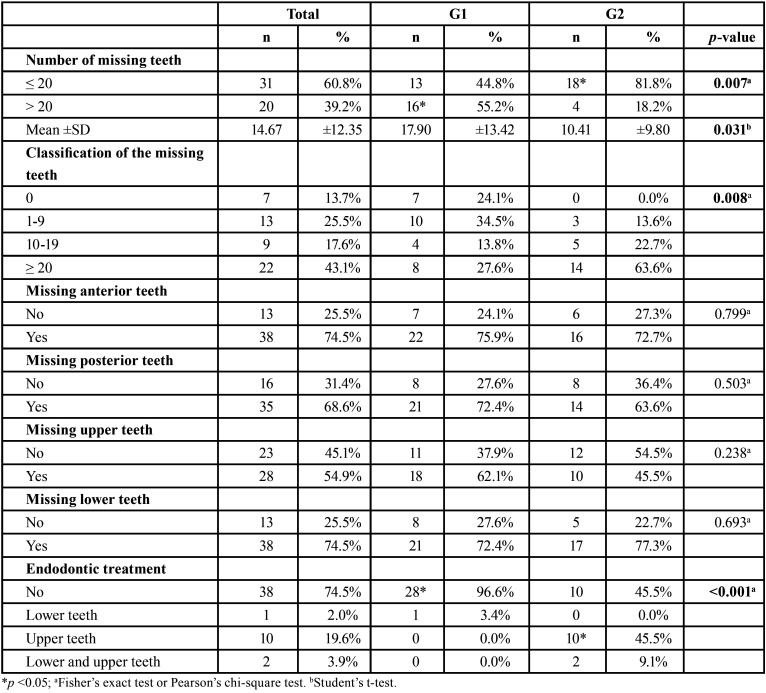
Comparison of dental status between groups.

-Biochemical data 

There was no statistical difference in the concentration of ionized and total calcium between the groups, but the mean phosphorus level was significantly lower in G1 (*p*=0.009). There was also no statistical difference in PTH, alkaline phosphatase, and creatinine levels between the groups; however, 24-hour urinary calcium (*p*=0.017) and vitamin D levels (*p* <0.001) were significantly higher in G1. In G1, 40% of the patients had already undergone surgery for pHPT, which was unsuccessful or did not restore metabolic control to maintain adequate parameters. During the dental evaluation, all patients still had untreated pHPT and were awaiting surgery since most of them were young and asymptomatic (53%). In G2, all were awaiting surgery.

-Clinical and systemic parameters

The mean age at diagnosis of pHPT in G1 was 31.55 ± 11.45 (17-61) years, whereas the mean age was 58.77 ± 11.72 (38-84) years in G2. The age at diagnosis of pHPT was significantly lower in G1 patients (*p*<0.001) (Table 3). The time elapsed between the diagnosis of pHPT and the oral examination performed in this study was 8.75 ± 7.61 years in G1, and 5.27 ± 6.58 years in G2. Regarding the bone mineral status, the frequency of patients that reported osteoporosis did not differ (*p*=0.780) between G1 (44.8%) and G2 (40.9%). Osteopenia was statistically significant in G2 (45.5%, *p*=0.029). G1 patients reported osteoporosis at a younger age than G2 patients (*p*=0.004). Patientis in G1 exhibited fractures (traumatic or spontaneous) at a younger age than G2 (*p*=0.013). There was no statistical difference between the groups regarding the prevalence of diabetes mellitus (*p*=0.861) and the use of antiresorptive agents (*p*=0.228). The use of oral calcium was significantly higher in G2 (*p*=0.002).

**Table 3:**
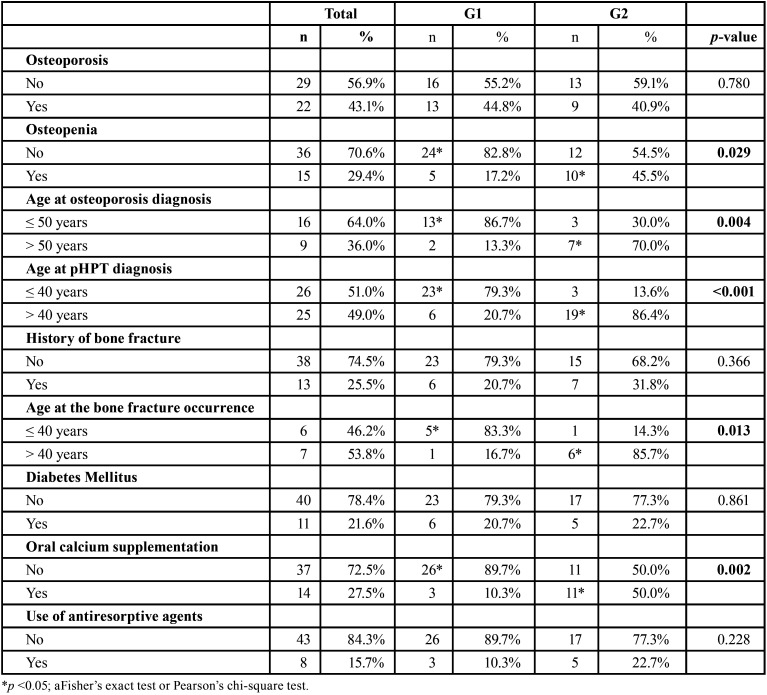
Comparison of osseous metabolism-related findings between groups.

## Discussion

In the present sample, there was a predominance of female individuals in both groups. Sporadic pHPT is known to more frequently affect females, especially after menopause; however, in MEN-1 cases, pHPT involvement occurs in both sexes in the same proportion. The predominance of female patients with MEN-1 observed in this study could also be a result of an increasing tendency of seeking medical care by women. The sociodemographic analysis showed that most patients were ≤50 years old, with a significantly higher prevalence of older individuals in G2, as it is the group of patients with sporadic pHPT. Diagnosis of hereditary pHPT associated with MEN-1 has been described at ages as early as 8 years, which according to the last consensus, should be followed by a biochemical screening for pHPT (10). In terms of the severity of pHPT the groups were similar, considering that we did not observe differences in the concentrations of calcium and PTH.

We also observed that most patients had ≤20 natural teeth in the oral cavity; however, despite having a more significant number of younger individuals, G1 had a higher frequency of tooth loss (>20 missing teeth), a greater number of missing upper teeth, higher frequency of teeth with active or inactive caries as well as enamel breakdown. As we did not observe any difference between the mean levels of PTH, ionized calcium, and total calcium between the groups, the severity of pHPT could not explain the difference observed. This fact does not appear to be associated with socioeconomic status or educational level, as there was no statistical difference between the groups when comparing these factors. A possible explanation could be that G1 patients have a more insidious and long-lasting disease (time between pHPT diagnosis and oral evaluation of 8.75 ± 7.61 years in G1 vs 5.27 ± 6.58 years in G2), which generates uncertainty regarding the period in which the PTH levels start to increase and its consequences on the quality of dentition and oral health.

The effect of HPT on oral health was evaluated in an Indian population (11) in which the authors during the radiological evaluation of patients with moderate to severe sporadic pHPT (serum PTH 769 ± 751 pg/ml (range 123 to 2139 pg/ml) observed that loss of lamina dura and mandibular cortical width are significantly correlated with plasma levels of PTH. In the present study, we found lower mean PTH levels compared to the above-mentioned study, suggesting that milder levels of PTH might explain the absence of radiological changes.

Our data demonstrated that periodontal involvement was a common finding in patients with pHPT. A previous study carried out in our population evaluated periodontal disease in patients with acromegaly (12), with no evidence of such involvement. The authors highlightes that the growth hormone has a protective effect on oral health because of its anabolic effect. However, another study carried out with patients with acromegaly demonstrated a high prevalence of gingival bleeding, being the most apparent characteristic of gingival inflammation (13).

In the present study, we observed that grade 2 dental mobility, which reflects the presence of periodontal disease or occlusal trauma, and other indicators of periodontal involvement (gingival bleeding index - grade 2, plaque index - grade 2, mean probing depth, loss of clinical insertion level and loss of gingival margin level) were significantly more prevalent in G1 patients. This finding also reveals that more significant periodontal changes were observed in the group consisting of younger patients. Thus, the duration of pHPT may be contributing to these findings since its autosomal dominant hereditary profile makes it is difficult to precisely identify the onset of metabolic changes. The contribution of PTH to this event is, however, questionable. In the literature, the results of a study with pHPT patients suggested that high levels of PTH cause a reduction of the cortical portion of the mandible and allows the expansion of trabecular bone because of its anabolic effect (4). Thus, PTH could also be involved in the development of periodontal disease.

Considering that poor oral hygiene and insufficient professional dental care can contribute to the development of periodontal disease, in our study, we observed a low frequency of brushing associated with low socioeconomic status, which reflects a population with limited access to dental care.

Hyposalivation was observed in the present study; however, no statistically significant difference was found between the groups. The presence of hyposalivation and high calcium concentrations can contribute to dental plaque accumulation and gingival calculus formation, negatively impacting oral health (23), as demonstrated by the gingival bleeding index (grade 2) and the plaque index (grade 2), which were higher in the group with hereditary HPT. Thus, our data demonstrate a decline in the oral health with hereditary pHPT despite most younger individuals.

G1 also had significantly more patients with tori, with the majority located on the palate. This condition has been reported to affect more women than men (ratio 2: 1) and can occur at any age, being, nevertheless, uncommon in early childhood. Furthermore, certain ethnic groups, such as American Indians and Eskimos, have high incidences of palatine tori (15). In a study in an Indian population with moderate to severe pHPT, without differentiation between the sporadic and the hereditary form, the authors found no patients with mandibular tori (16). However, the authors questioned its relationship with pHPT and suggested further studies with a larger number of individuals. The authors also questioned if a more severe pHPT could contribute to a more significant loss of both cortical and trabecular bone, thus reducing the formation and expansion of the trabecular bone. In contrast, Padbury *et al*., 2006 (4) demonstrated a high prevalence of mandibular tori in individuals with pHPT, which is relatively similar to the findings of our study, demonstrating an increased relative frequency of palatine tori. The presence of mandibular tori is determined by multiple factors such as genetic and environmental variables, including occlusal forces. Another mechanism recently proposed to explain the occurrence of oral tori is the combination of biomechanical forces, mainly from the oral cavity, with cortical bone loss and trabecular expansion. This predominant loss of cortical bone and increased formation of trabecular bone usually occurs in the early stages of pHPT (16). Thus, as G1 consisted of individuals who theoretically had a more prolonged asymptomatic stage and more insidious disease, our findings may reflect the degree of cortical bone loss and increased trabecular bone formation, especially in the palate region when compared to the mandibular region.

## Conclusions

The present study introduces relevant findings concerning the oral health of individuals with pHPT. Patients with hereditary pHPT, although usually younger, consistently presented diminished oral health indicators. Periodontal involvement, tori presence, dentition changes, and tooth loss when observed in young individuals, which could be observed during routine visits to the dentist, may contribute to an early diagnosis of pHPT. The severity of pHPT only or socioeconomic status only does not explain these findings, thereby requiring a methodological assessment of clinical, radiographic, and biochemical parameters. In the present research, the interdisciplinary approach to be conducted by general dentists and endocrinologists was emphasized, which could help reduce comorbidities and benefit patients with various forms of HPT.
